# Chemical Constituents from the Leaves of *Annona reticulata* and Their Inhibitory Effects on NO Production

**DOI:** 10.3390/molecules18044477

**Published:** 2013-04-16

**Authors:** Tran Dinh Thang, Ping-Chung Kuo, Guan-Jhong Huang, Nguyen Huy Hung, Bow-Shin Huang, Mei-Lin Yang, Ngo Xuan Luong, Tian-Shung Wu

**Affiliations:** 1 Department of Chemistry, Vinh University, Vinh 42000, Vietnam; E-Mail: thangtd@vinhuni.edu.vn (T.D.T.); 2 Department of Biotechnology, National Formosa University, Yunlin 632, Taiwan; E-Mail: pcckuoo@sunws.nfu.edu.tw (P.-C.K.); 3 Department of Chinese Pharmaceutical Sciences and Chinese Medicine Resources, China Medical University, Taichung 404, Taiwan; 4 Department of Chemistry, National Cheng Kung University, Tainan 701, Taiwan; 5 Department of Natural Science, Hong Duc University, Thanhhoa 41000, Vietnam

**Keywords:** *Annona*, triterpenoid, diterpenoid, NO inhibition, *i*NOS, macrophage

## Abstract

In the present study, the chemical investigation of the leaves of *Annona reticulata* has resulted in the identification of nine compounds, including annonaretin A, (**1**), a new triterpenoid. The purified compounds were subjected to the examination of their effects on NO inhibition in LPS-activated mouse peritoneal macrophages and most of them exhibited significant NO inhibition, with IC_50_ values in the range of 48.6 ± 1.2 and 99.8 ± 0.4 μM.

## 1. Introduction

The *Annona* genus (Annonaceae) consists of about 119 species, most of which are shrubs and trees widely distributed in the tropical and subtropical regions, including the Southeast Asia countries such as Malaysia, Indonesia, Thailand, Cambodia, Laos, and Vietnam. In Indian folk medicine, various species of *Annona* have been used as vermifuges, anti-inflammatory agents, in wound healing, as antimalarial agents and in the treatment of diarrhoea and dysentery [1]. The bark of the plant *Annona reticulata* L. is a powerful astringent and given as tonic. The plant has been used as an anti-inflammatory agent in wound healing, anti-anxiety, anti-stress, anti-mutagenic, and spasmolytic agent. Leaf and stem extract shows inotropic, positive chronotropic and spasmolytic activities [2]. The plant is reported to contain acetogenins mainly *cis*- and *trans*-isomurisolenin [3], annoreticuin, bullatacin, squamosine, rolliniastatin [4], reticullacinone, rolliniastatin-2, molvizarin [5], 14-hydroxy-25-deoxy-rollinicin [6]. Reticulatacin and kaurane diterpenes were also identified from the bark of the plant [7]. Other terpenes such as spathenelol, muurolene, copaene and eudesmol were reported in the previous literature [8]. In our continued program aimed at the identification of anti-inflammatory drug leads from natural sources, the chemical composition of the leaves of *A. reticulata* was investigated to search for the bioactive constituents by assays of inhibitory effects on NO inhibition in LPS-activated mouse peritoneal macrophages. In the present study, we wished to report the characterization of nine compounds, including the structural determination of new compound **1**, as well as their NO inhibitory effects.

## 2. Results and Discussion

### 2.1. Isolation and Characterization of Compounds

**Figure 1:**
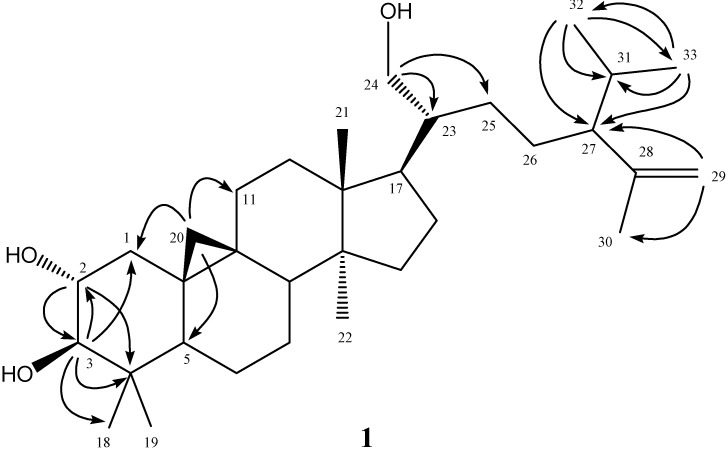
Structure and significant HMBC (→) correlations of compound **1**.

**Figure 2:**
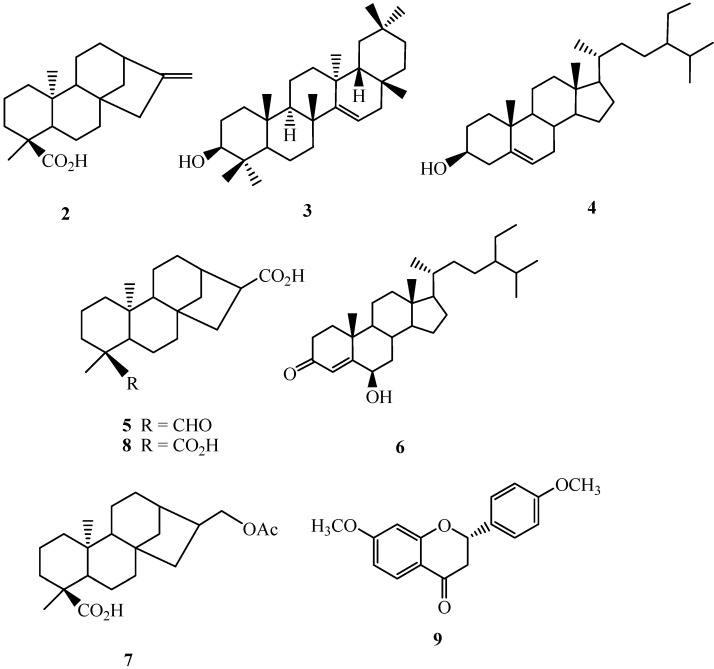
Structures of purified known compounds **2**–**9**.

Air-dried and powdered leaves of *A. reticulata* L. were soaked with methanol at room temperature, and the combined extracts were concentrated to give a deep brown syrup. The crude extract was suspended into water and partitioned with *n*-hexane, ethyl acetate, and *n*-butanol, successively to afford *n*-hexane, ethyl acetate, *n*-butanol, and water solubles, respectively. Purification of the *n*-hexane soluble fraction by column chromatography yielded annonaretin A (**1**) (Figure 1), kaurenoic acid (**2**) [9], taraxerol (**3**) [10], β-sitosterol (**4**) [11], 16α-hydro-19-al-*ent*-kauran-17-oic acid (**5**) [9], 6β-hydroxystigmast-4-en-3-one (**6**) [12], and 17-acetoxy-16β-*ent*-kauran-19-oic acid 24 (**7**) [13]. Purification of the ethyl acetate soluble afforded 16α-hydro-*ent*-kauran-17,19-dioic acid (**8**) [9], and (2*S*)-di-*O*-methylquiritigenin (**9**) [14]. Compounds **2**–**9** (Figure 2) are known compounds, and their structures were identified by comparison of their physical and spectroscopic data with those reported in the literature. Compound **1** is new. 

### 2.2. Structural Elucidation of New Compound ***1***

The purified colorless powder **1** was visualized by spraying with 1% (w/v) Ce(SO_4_)_2_ in 10% (v/v) aqueous H_2_SO_4_ followed by heating at 120 °C and displayed purplish black spots on TLC plate. It also displayed a positive response in the Lieberman–Burchard test. These results suggested that compound **1** possessed a basic triterpenoid skeleton [15]. The molecular formula of **1** was established as C_33_H_56_O_3_ by the pseudomolecular [M+Na]^+^ ion peak at *m/z* 523.4122 in HR-ESI-MS analysis and was further supported by its ^13^C-NMR spectrum which showed signals for all 33 carbons of the molecule, including one set of terminal methylenes (δ_C_ 111.9, 147.4), three oxygenated carbons (δ_C_ 62.8, 71.0, 83.2), and seven methyl carbons (δ_C_ 15.1, 18.1, 19.0, 19.3, 20.7, 21.3, 25.7). In the ^1^H-NMR spectrum of **1**, there were five singlets at δ 0.82 (3H, s, CH_3_-18), 0.91 (3H, s, CH_3_-21), 0.97 (3H, s, CH_3_-22), 0.98 (3H, s, CH_3_-19), and 1.60 (3H, s, CH_3_-30); and two doublets at δ 0.80 (3H, d, *J* = 9.5 Hz, CH_3_-32) and 0.93 (3H, d, *J* = 6.0 Hz, CH_3_-33), respectively. In addition, two high field cyclopropyl proton doublets at δ 0.40 (1H, d, *J* = 4.5 Hz, H-20) and 0.62 (1H, d, *J* = 4.5 Hz, H-20) evidenced that this compound was a triterpenoid derivative with the cycloartanol skeleton. In the downfield region, four oxygenated protons at δ 3.01 (1H, d, *J* = 9.5 Hz, H-3), 3.54 (1H, dd, *J* = 11.0, 6.0 Hz, H-24), 3.64 (1H, br dd, *J* = 16.0, 9.0 Hz, H-2), and 3.70 (1H, br d, *J* = 11.0 Hz, H-24) were located at C-2, -3, and -24 determined by the ^2^*J*, ^3^*J*-HMBC correlations through H-2 to C-3 (δ 83.2), C-4 (δ 40.3); H-3 to C-1 (δ 25.7), C-2 (δ 71.0), C-4, and C-18 (δ 15.1); and H-24 to C-23 (δ 46.2), C-25 (δ 27.7), respectively. The position of terminal methylene group at δ 4.64 (1H, br s, H-29) and 4.77 (1H, br s, H-29) was also establsihed with the HMBC analysis of correlations between H-29 and C-27 (δ 55.4), C-30 (δ 19.0). In the HMBC spectral analysis (Figure 2), correlation peaks between H-32 and C-33, C-31, C-27; H-33 and C-32, C-31, C-27, supported that one isopropyl group was attached at C-27. The high field doublets at δ 0.40 and 0.62 displayed HMBC correlations with the carbons at δ 25.7 (C-1), 39.7 (C-11), and 47.1 (C-5) also confirmed the presence of cyclopropyl functionality as C-20 connected with C-9 and C-10. The full assignments of ^1^H- and ^13^C-NMR signals were substantiated by extensive 2D NMR experimental analysis. Therefore the chemical structure of **1** was established as shown in Figure 1 and the compound was given the trivial name annonaretin A.

### 2.3. The Inhibitory Effects of Isolated Compounds on NO Production

The isolated compounds **1**–**9** were subjected into the examination of their effects on LPS-induced *i*NOS-dependent NO production in RAW 264.7 cells. Cells cultured with **1**–**9** at different concentrations used in the presence of 100 ng/mL LPS for 24 h (Table 1). Some cell toxicity was observed in cells treated with compounds **1**, **2**, **4**, and **9**, whereas other compounds had no influence on cell viability. NO production was significantly decreased by the treatment with **2**, **3**, and **5**–**8** in a dose-dependent manner, with IC_50_ values in the range of 48.6 ± 1.2 and 99.8 ± 0.4 μM (Table 1). The inhibitory effects of **1**, **4** and **9** were less obvious. **2**, **3**, and **5**–**8** did not exhibit significant cytotoxicity in the concentration range of 12.5–100 μM (Table 1), thus the NO inhibiting effects were probably not due to cytotoxicity. Among the tested compounds, **2**, and **5–8** belonged to the *ent*-kaurane diterpenoids noted for the occurrence in the *Annona* species [16,17]. In the previous literature, kaurenoic acid (**2**) was reported to inhibit NO production, prostaglandin E2 release, cyclooxygenase-2, and inducible nitric oxide synthase expression in LPS-induced RAW264.7 macrophages [18]. It also exhibited anti-inflammatory [19,20], cytotoxic [21,22], antiplasmodial [21], antimicrobial [16], hypoglycaemic [23], vasorelaxant [24,25], and antispasmodic [25] bioactivities. 16α-Hydro-19-al-*ent*-kauran-17-oic acid (**5**) displayed the most significant inhibitory effect on NO production with the lowest IC_50_ value of 48.6 ± 1.2 μM. It was also reported from the stems of *A. squamosa* to exhibit anti-platelet aggregation activity [26]. In addition, taraxerol (**3**) could downregulate the expression of proinflammatory mediators in macrophages by interfering with the activation of TAK1 and Akt, thus preventing NF-κB activation [27]. In our experimental results, **2**, **3**, and **5** displayed NO inhibitory effects similar to these anti-inflammatory reports.

**Table 1:** Inhibitory effects of purified samples from the leaves of *A. reticulata* on LPS-induced *i*NOS-dependent NO production in RAW 264.7 cells.

****	**Dose (μM)**	**Cell viability(% of control)**	**NO level**	**NO inhibition(% of control)**	**IC_50_(μM)**
Control	(−)	100.0 ± 4.9	−0.5 ± 0.1	(−)	(−)
LPS	(+)	98.7 ± 8.0	45.4 ± 2.7 ^###^	(−)	(−)
**1**	12.5 25 50 100	88.0 ± 1.5 81.2 ± 1.8 73.0 ± 3.6 * 66.9 ± 5.5 **	36.6 ± 1.9 33.5 ± 0.9 (−) (−)	19.3 ± 4.2 26.2 ± 2.0 (−) (−)	(−)
**2**	12.5 25 50 100	98.0 ± 2.2 91.0 ± 3.6 82.9 ± 1.2 65.7 ± 8.6 **	34.3 ± 4.7 35.1 ± 1.2 22.7 ± 0.5 ** (−)	24.5 ± 10.2 22.7 ± 2.6 50.0 ± 1.0 (−)	50.0 ± 0.3
**3**	12.5 25 50 100	93.5 ± 1.8 92.6 ± 3.1 88.7 ± 1.8 81.4 ± 2.3	38.5 ± 2.3 39.5 ± 0.6 35.6 ± 0.9 * 22.6 ± 0.4 **	15.2 ± 5.0 13.1 ± 1.2 21.6 ± 2.0 50.1 ± 0.9	99.8 ± 0.4
**4**	12.5 25 50 100	82.8 ± 2.7 80.7 ± 3.8 65.3 ± 2.3 ** 55.8 ± 2.7 **	38.4 ± 0.7 38.6 ± 3.3 (−) (−)	15.5 ± 1.5 15.1 ± 7.3 (−) (−)	(−)
**5**	12.5 25 50 100	98.9 ± 5.2 96.5 ± 9.2 95.9 ± 8.6 80.2 ± 5.7	38.3 ± 1.3 33.1 ± 1.0 * 22.1 ± 1.5 ** 14.8 ± 0.7 ***	15.5 ± 2.8 27.1 ± 2.3 51.4 ± 3.2 67.3 ± 1.5	48.6 ± 1.2
**6**	12.5 25 50 100	94.8 ± 3.1 83.7 ± 3.5 83.5 ± 3.0 81.3 ± 2.8	37.0 ± 1.8 33.1 ± 2.4 * 22.9 ± 1.1 ** 15.1 ± 0.3 ***	18.5 ± 4.0 27.1 ± 5.3 49.5 ± 2.4 66.7 ± 0.6	51.5 ± 0.5
**7**	12.5 25 50 100	98.6 ± 3.1 93.6 ± 4.0 92.5 ± 2.8 83.7 ± 1.6	42.6 ± 0.7 40.6 ± 2.8 23.7 ± 0.2 ** 14.3 ± 0.3 ***	6.2 ± 1.5 10.6 ± 6.1 47.7 ± 0.3 68.5 ± 0.7	55.5 ± 0.3
**8**	12.5 25 50 100	98.4 ± 3.2 94.0 ± 2.6 88.6 ± 2.6 82.4 ± 1.2	39.8 ± 1.2 35.4 ± 0.8 * 23.5 ± 0.9 ** 14.4 ± 0.3 ***	12.3 ± 2.6 21.9 ± 1.8 48.2 ± 2.0 68.3 ± 0.7	54.5 ± 0.8
**9**	12.5 25 50 100	99.9 ± 4.8 90.0 ± 1.6 82.6 ± 2.7 76.2 ± 1.1 *	42.0 ± 1.2 40.4 ± 0.7 25.9 ± 2.3 (−)	7.5 ± 2.7 11.0 ± 1.6 42.9 ± 5.0 (−)	(−)

The data were presented as mean ± S.D. ^###^ Compared with sample of control group. * *p* < 0.05, ** *p* < 0.01, and *** *p* < 0.001 were compared with LPS-alone group. (−): Not determined.

## 3. Experimental

### 3.1. General Procedures

Melting point was determined using an Electrothermal IA-9200 melting point measuring apparatus without correction. The UV spectrum was recorded on an Agilent UV-VIS recording spectrophotometer. The IR spectra (KBr) were obtained, on a Hitachi 270-30 type spectrometer. Optical rotations were measured with a Jasco DIP-1000 KUY polarimeter. The electrospray ionization (ESI) mass spectra were determined using an Agilent 1200 LC-MSD Trap spectrometer, and the HR-ESI-MS was completed with the aid of a Bruker APEX II mass spectrometer. ^1^H- and ^13^C-NMR, COSY, NOESY, HMQC, and HMBC spectra were recorded on a Bruker Avance-500 NMR spectrometer, using tetramethylsilane (TMS) as the internal standard. Standard pulse sequences and parameters were used for the NMR experiments and all chemical shifts were reported in parts per million (ppm, δ). Column chromatography (CC) was performed on silica gel (Kieselgel 60, 70–230 mesh and 230–400 mesh, E. Merck, Darmstadt, Germany).

### 3.2. Plant Materials

The leaves of *Annona reticulata* L. (Annonaceae) were collected from Tiengiang, Vietnam, during October 2010 and the plant materials were identified and authenticated by Dr. Tran Huy Thai, Institute of Ecology and Biological Resources, Vietnamese Academy of Science and Technology. A voucher specimen (Viet-TSWu-20101015) was deposited at the Herbarium of the Vinh University.

### 3.3. Extraction and Isolation

The leaves of *Annona reticulata* L. (5.0 kg) were air-dried and powdered and soaked (24 h) with methanol (20 L × 3) at room temperature, and the combined extracts were concentrated under reduced pressure to give deep brown syrup (316.0 g). The crude extract was suspended into water (1 L) and partitioned with *n*-hexane (1 L × 5), ethyl acetate (1 L × 5), and *n*-butanol (1 L × 5), successively to afford *n*-hexane (31.0 g), ethyl acetate (82.0 g), *n*-butanol (47.0 g), and water solubles (52.0 g), respectively, after removal of the corresponding solvent. 

The *n*-hexane soluble extracts were purified by silica gel column chromatography eluted with *n*-hexane and acetone gradients to afford 14 fractions. Fraction 1 was subjected to silica gel column chromatography eluted with *n*-hexane/acetone (25:1) to yield kaurenoic acid (**2**, 968 mg). Fraction 2 was isolated by silica gel column chromatography eluted with *n*-hexane/acetone (15:1) to afford taraxerol (**3**, 78 mg). Purification of fraction 3 by column chromatography with silica gel eluted by step gradients of *n*-hexane/acetone (15:1 and 9:1) afforded β-sitosterol (**4**, 302 mg). Fraction 4 was subjected to silica gel column chromatography eluted with *n*-hexane/acetone (15:1) to yield 16α-hydro-19-al-*ent*-kauran-17-oic acid (**5**, 26 mg). Fraction 5 was purified by silica gel column chromatography eluted with *n*-hexane/acetone (9:1) to yield 6*β*-hydroxystigmast-4-en-3-one (**6**, 31 mg). Isolation of fraction 6 by column chromatography with silica gel eluted by *n*-hexane/acetone (7:1) yielded 17-acetoxy-16β-*ent*-kauran-19-oic acid (**7**, 22 mg). Silica gel column chromatography of fraction 9 with step gradient elution of *n*-hexane/acetone (7:1 and 5:1) led to the purification of annonaretin A (**1**, 789 mg).

The ethyl acetate soluble extracts were applied to silica gel column chromatography with chloroform and methanol gradients (20:1 to 5:1) to afford eight fractions. Fraction 1 was subjected to silica gel column chromatography eluted with *n*-hexane/acetone (9:1) to yield 16α-hydro-*ent*-kauran-17,19-dioic acid (**8**, 38 mg). Silica gel column chromatography of fraction 6 with step gradient elution of chloroform and methanol (9:1 and 5:1) led to the isolation of (2*S*)-di-*O*-methylquiritigenin (**9**, 23 mg).

### 3.4. Spectral Data

Annonaretin A (**1**): colorless powder; m.p. 120−121 °C (CHCl_3_); ^1^H-NMR (CDCl3) δ 0.40 (1H, d, *J* = 4.5 Hz, H-20), 0.62 (1H, d, *J* = 4.5 Hz, H-20), 0.78 (1H, m, H-6), 0.80 (3H, d, *J* = 9.5 Hz, CH_3_-32), 0.82 (3H, s, CH_3_-18), 0.91 (3H, s, CH_3_-21), 0.93 (3H, d, *J* = 6.0 Hz, CH_3_-33), 0.97 (3H, s, CH_3_-22), 0.98 (3H, s, CH_3_-19), 1.04 (1H, m, H-7), 1.10–1.20 (3H, m, H-1, -25, -16), 1.31–1.43 (7H, m, H-1, -12, -26, -5, -17, -25), 1.52–1.69 (9H, m, H-31, -11, -8, -15, -27, -6, -30, -16), 1.60 (3H, s, CH_3_-30), 1.86–2.05 (3H, m, H-23, -26, -7), 3.01 (1H, d, *J* = 9.5 Hz, H-3), 3.40 (1H, br s, OH), 3.54 (1H, dd, *J* = 11.0, 6.0 Hz, H-24), 3.64 (1H, br dd, *J* = 16.0, 9.0 Hz, H-2), 3.70 (1H, br d, *J* = 11.0 Hz, H-24), 4.64 (1H, br s, H-29), 4.77 (1H, br s, H-29); ^13^C-NMR (CDCl3) δ 15.1 (C-18), 18.1 (C-19), 19.0 (C-30), 19.2 (C-10), 19.3 (C-21), 20.7 (C-33), 20.9 (C-6), 21.3 (C-32), 25.1 (C-9), 25.7 (C-22), 25.7 (C-1), 26.5 (C-7), 26.8 (C-16), 27.3 (C-26), 27.7 (C-25), 29.7 (C-20), 30.2 (C-31), 31.8 (C-15), 35.3 (C-12), 39.7 (C-11), 40.3 (C-4), 43.1 (C-17), 45.0 (C-13), 46.2 (C-23), 47.1 (C-5), 47.7 (C-8), 48.7 (C-14), 55.4 (C-27), 62.8 (C-24), 71.0 (C-2), 83.2 (C-3), 111.9 (C-29), 147.4 (C-28); ESI-MS *m/z* (*rel. int.*) 523 ([M+Na]^+^, 100); HR-ESI-MS *m/z* 523.4122 [M+Na]^+^ (calcd for C_33_H_56_O_3_Na, 523.4127).

### 3.5. Determination of Inhibitory Effects on NO Production

#### 3.5.1. Cell Culture

A murine macrophage cell line RAW264.7 (BCRC No. 60001) was purchased from the Bioresources Collection and Research Center (BCRC) of the Food Industry Research and Development Institute (Hsinchu, Taiwan). Cells were cultured in plastic dishes containing Dulbecco’s Modified Eagle Medium (DMEM, Sigma, St. Louis, MO, USA) supplemented with 10% fetal bovine serum (FBS, Sigma, USA) in a CO_2_ incubator (5% CO_2_ in air) at 37°C and subcultured every 3 days at a dilution of 1:5 using 0.05% trypsin–0.02% EDTA in Ca^2+^-, Mg^2+^- free phosphate-buffered saline (DPBS).

#### 3.5.2. Cell Viability

Cells (2 × 10^5^) were cultured in 96-well plate containing DMEM supplemented with 10% FBS for 1 day to become nearly confluent. Then cells were cultured with samples in the presence of 100 ng/mL LPS for 24 h. After that, the cells were washed twice with DPBS and incubated with 100 μL of 0.5 mg/mL MTT for 2 h at 37 °C testing for cell viability. The medium was then discarded and 100 μL dimethyl sulfoxide (DMSO) was added. After 30-min incubation, absorbance at 570 nm was read using a microplate reader (Molecular Devices, Sunnyvale, CA, USA).

#### 3.5.3. Measurement of Nitric oxide/Nitrite

NO production was indirectly assessed by measuring the nitrite levels in the cultured media and serum determined by a colorimetric method based on the Griess reaction [28]. The cells were incubated with a test sample in the presence of LPS (100 ng/mL) at 37 °C for 24 h. Then, cells were dispensed into 96-well plates, and 100 μL of each supernatant was mixed with the same volume of Griess reagent (1% sulfanilamide, 0.1% naphthyl ethylenediamine dihydrochloride, and 5% phosphoric acid) and incubated at room temperature for 10 min, the absorbance was measured at 540 nm with a Micro-Reader (Molecular Devices). By using sodium nitrite to generate a standard curve, the concentration of nitrite was measured form absorbance at 540 nm.

#### 3.5.4. Statistical Analysis

Experimental results were presented as the mean ± standard deviation (SD) of three parallel measurements. Statistical comparisons were made by Student’s t-test. IC_50_ values were estimated using a non-linear regression algorithm (SigmaPlot 8.0; SPSS Inc. Chicago, IL, USA). Statistical significance is expressed as * *p* < 0.05, ** *p* < 0.01, and *** *p* < 0.001.

## 4. Conclusion

In summary, nine compounds were characterized from the leaves of *A. reticulata* L. and their inhibitory activity on NO production was examined. The results provide a potential explanation for the use of the leaves of *A. reticulata* as a herbal medicine in the treatment of inflammatory diseases, and they may be potentially useful in developing new anti-inflammatory agents.

## References

[1] Kirtikar K.R., Basu B.D. (1987). Indian Medicinal Plants.

[2] (1994). The Useful Plants of India.

[3] Chang F.R., Chen J.L., Chiu H.F., Wu M.J., Wu Y.C. (1998). Acetogenins from seeds of *Annona reticulata*. Phytochemistry.

[4] Maeda U., Hara N., Fujimoto Y., Shrivastava A., Gupta Y.K., Sahai M. (1993). *N*-fatty acyl tryptamines from *Annona reticulata*. Phytochemistry.

[5] Hisham A., Sunitha C., Sreekala U., Pieters L., De Bruyne T., Van den Heuvel H., Claeys M. (1994). Reticulacinone, an acetogenin from *Annona reticulata*. Phytochemistry.

[6] Etse J.T., Waterman P.G. (1986). Chemistry in the Annonaceae, XXII. 14-Hydroxy-25-desoxyrollinicin from the stem bark of *Annona reticulata*. J. Nat. Prod..

[7] Saad J.M., Hui Y.H., Rupprecht J.K., Anderson J.E., Kozlowski J.F., Zhao G.X., Wood K.V., McLaughlin J.L. (1991). Reticulatacin: A new bioactive acetogenin from *Annona reticulata* (Annonaceae). Tetrahedron.

[8] Jirovetz L., Buchbauer G., Shafi P.M., Saidutty A. (1998). Analysis of the essential oils of the leaves and roots of *Annona reticulata* from South-India. Ernaehrung.

[9] Hsieh T.J., Wu Y.C., Chen S.C., Huang C.S., Chen C.Y. (2004). Chemical constituents from *Annona glabra*. J. Chin. Chem. Soc..

[10] Kanlayavattanakul M., Ruangrungsi N., Watanabe T., Kawahata M., Therrien B., Yamaguchi K., Ishikawa T. (2005). *ent*-Halimane diterpenes and a guaiane sesquiterpene from *Cladogynos orientalis*. J. Nat. Prod..

[11] Nes W.D., Norton R.A., Benson M. (1992). Carbon-13-NMR studies on sitosterol biosynthesized from [^13^C]mevalonates. Phytochemistry.

[12] Kuo Y.H., Chu P.H. (2002). Studies on the constituents from the bark of *Bauhinia purpurea*. J. Chin. Chem. Soc..

[13] Chen C.Y., Chang F.R., Wu Y.C. (1997). The constituents from the stems of *Annona cherimola*. J. Chin. Chem. Soc..

[14] Yoshida T., Feng W.S., Okuda T. (1993). Two polyphenol glycosides and tannins from *Rosa cymosa*. Phytochemistry.

[15] Srivastava A., Shukla Y.N. (1998). Aryl esters and a coumarin from *Aygyreia speciosa*. Indian J. Chem. Sect. B.

[16] Okoye T.C., Akah P.A., Okoli C.O., Ezike A.C., Omeje E.O., Odoh U.E. (2012). Antimicrobial effects of a lipophilic fraction and kaurenoic acid isolated from the root bark extracts of *Annona senegalensis*. *Evid*. *Based Complement*. Alternat. Med..

[17] Guillopé R., Escobar-Khondiker M., Guérineau V., Laprévote O., Höglinger G.U., Champy P. (2011). Kaurenoic acid from pulp of *Annona cherimolia* in regard to Annonaceae-induced Parkinsonism. Phytother. Res..

[18] Choi R.J., Shin E.M., Jung H.A., Choi J.S., Kim Y.S. (2011). Inhibitory effects of kaurenoic acid from *Aralia continentalis* on LPS-induced inflammatory response in RAW264.7 macrophages. Phytomedicine.

[19] Mizokami S.S., Arakawa N.S., Ambrosio S.R., Zarpelon A.C., Casagrande R., Cunha T.M., Ferreira S.H., Cunha F.Q., Verri W.A. (2012). Kaurenoic acid from *Sphagneticola trilobata* inhibits inflammatory pain: Effect on cytokine production and activation of the NO-cyclic GMP-protein kinase G-ATP-sensitive potassium channel signaling pathway. J. Nat. Prod..

[20] Lim H., Jung H.A., Choi J.S., Kim Y.S., Kang S.S., Kim H.P. (2009). Anti-inflammatory activity of the constituents of the roots of *Aralia continentalis*. Arch. Pharm. Res..

[21] Batista R., García P.A., Castro M.A., Miguel Del Corral J.M., Speziali N.L., de P Varotti F., de Paula R.C., García-Fernández L.F., Francesch A., San Feliciano A. (2013). Synthesis, cytotoxicity and antiplasmodial activity of novel *ent*-kaurane derivatives. Eur. J. Med. Chem..

[22] De Andrade B.B., Moreira M.R., Ambrosio S.R., Furtado N.A., Cunha W.R., Heleno V.C., Silva A.N., Simão M.R., Da Rocha E.M., Martins C.H. (2011). Evaluation of *ent*-kaurenoic acid derivatives for their anticariogenic activity. Nat. Prod. Commun..

[23] Raga D.D., Alimboyoguen A.B., Del Fierro R.S., Ragasa C.Y. (2010). Hypoglycaemic effects of tea extracts and *ent*-kaurenoic acid from *Smallanthus sonchifolius*. Nat. Prod. Res..

[24] Tirapelli C.R., Ambrosio S.R., da Costa F.B., Coutinho S.T., de Oliveira D.C., de Oliveira A.M. (2004). Analysis of the mechanisms underlying the vasorelaxant action of kaurenoic acid in the isolated rat aorta. Eur. J. Pharmacol..

[25] Ambrosio S.R., Tirapelli C.R., Coutinho S.T., de Oliveira D.C., de Oliveira A.M., da Costa F.B. (2004). Role of the carboxylic group in the antispasmodic and vasorelaxant action displayed by kaurenoic acid. J. Pharm. Pharmacol..

[26] Yang Y.L., Chang F.R., Wu C.C., Wang W.Y., Wu Y.C. (2002). New *ent*-kaurane diterpenoids with anti-platelet aggregation activity from *Annona squamosa*. J. Nat. Prod..

[27] Yao X., Li G., Bai Q., Xu H., Lü C. (2013). Taraxerol inhibits LPS-induced inflammatory responses through suppression of TAK1 and Akt activation. Int.Immunopharmacol..

[28] Chang C.T., Huang S.S., Lin S.S., Amagaya S., Ho H.Y., Hou W.C., Shie P.H., Wu J.B., Huang G.J. (2011). Anti-inflammatory activities of tormentic acid from suspension cells of *Eriobotrya japonica ex vivo* and *in vivo*. Food Chem..

